# Development of Designed Site-Directed Pseudopeptide-Peptido-Mimetic Immunogens as Novel Minimal Subunit-Vaccine Candidates for Malaria

**DOI:** 10.3390/molecules15128856

**Published:** 2010-12-06

**Authors:** José Manuel Lozano, Liliana P. Lesmes, Luisa F. Carreño, Gina M. Gallego, Manuel Elkin Patarroyo

**Affiliations:** Fundación Instituto de Inmunología de Colombia (FIDIC), Universidad del Rosario and Universidad Nacional de Colombia, Bogotá DC, Colombia

**Keywords:** peptide high binding motif, peptide-bond isoster, transition-state analogue, pseudopeptide, Ig passive transferring, malaria vaccine

## Abstract

Synthetic vaccines constitute the most promising tools for controlling and preventing infectious diseases. When synthetic immunogens are designed from the pathogen native sequences, these are normally poorly immunogenic and do not induce protection, as demonstrated in our research. After attempting many synthetic strategies for improving the immunogenicity properties of these sequences, the approach consisting of identifying high binding motifs present in those, and then performing specific changes on amino-acids belonging to such motifs, has proven to be a workable strategy. In addition, other strategies consisting of chemically introducing non-natural constraints to the backbone topology of the molecule and modifying the α-carbon asymmetry are becoming valuable tools to be considered in this pursuit. Non-natural structural constraints to the peptide backbone can be achieved by introducing peptide bond isosters such as reduced amides, partially retro or retro-inverso modifications or even including urea motifs. The second can be obtained by strategically replacing L-amino-acids with their enantiomeric forms for obtaining both structurally site-directed designed immunogens as potential vaccine candidates and their Ig structural molecular images, both having immuno-therapeutic effects for preventing and controlling malaria.

## Abbreviations

HABP: High activity binding peptide
MHC: major histocompatibility complex
TCR: T-cell receptor
TCC: T-cell clone
APC: antigen presenting cell
CDR: complimentarily determinant region
HLA: human leukocyte antigens
IL-4: interleulin-4
INF-γ: gamma interferon
mAb: monoclonal antibody
MSA-1 or MSP-1: Merozoite Surface Antigen-1
MSA-2 or MSP-2: Merozoite Surface Antigen-2
rMSP-1: recombinant merozoite surface protein-1 fragment
RBC: red blood cells
iRBC: infected red blood cell
CSP: Circumsporozoite Surface Protein
IgM: immunoglobulin-M isotype
IgG: immunoglobulin-G isotype
F(ab)_2_’: immunoglobulins antigen binding fragment-2
NaBH(OAc)_3_: triacetoxyborohydride
DMF: *N,N’*-dimethylformamide
DCE: dichloro-ethane
THF: tetrahydrofuran
NaCNBH_3_: sodium cyanoborohydride
Pd/C: palladium over charcoal
4-MBHA: 4-methylbenzhydrylamine
*t*-Boc: *tert*-butyloxycarbonyl
DIEA: 9-fluorenylmethyloxycarbonyl
Fmoc: diisopropylethylamine
TFA: trifluoroacetic acid
TFE: trifluoroethanol
m-*R,S*-TMD: 2,2,5-trimethyl-1,3-dioxane-4,6-dione
RP-HPLC: reverse phase-high performance liquid chromatography
[D6]DMSO: deuterated-dimethylsulfoxide
^1^H-NMR: proton-nuclear magnetic resonance
CD: Circular Dichroism
NOE: Nuclear Overhauser effect
SPf-66: Synthetic Plasmodium falciparum-66 vaccine

## 1. Introduction

Specific antigenic determinants from pathogen proteins and peptides fit into specific receptors, known as major histocompatibility complexes (MHC); in this context, peptides are thus the target of immune recognition by T cells. Class I MHC bound peptides are presented to cytotoxic T-cells, while class II-MHC molecules bound peptides are presented to helper T-cells. On the other hand, peptide antagonists or partial agonists mimic the conformation of a known antigenic peptide and thereby maintain a satisfactory binding affinity for a particular MHC allotype. However, they differ sufficiently from the original peptide to change T cell signaling when the MHC-peptide complex interacts with TCR, being the peptide size and its structure important requirements for a suitable immune response.

During the last two decades, our laboratory has acquired experience and accumulated important evidence by testing 20-mer peptide synthetic polymers as sub-unit malaria vaccine candidates *in vitro,* as well as testing them *in vivo*. The role of reduced amide pseudopeptide analogues derived from malarial MSP-1 and MSP-2 antigens has been also assessed in both *in vitro* and *in vivo* studies. Our evidence suggest that these site-directed designed pseudopeptides represent conformational B-cell epitopes, capable of mimicking possible transient structural antigen states by modulating the molecule backbone, and also modulating immune response in animal models. In this review, we describe the development of the family of modified peptides (Reduced Amide Pseudopeptides) and also the impact of α-carbon asymmetrical modifications on the given malarial antigens, and their place within the wider realm of peptide modification.

Pseudopeptides thus strategically represent analogues, when the concept of “a bioactive conformation” has encouraged the design of molecules able to favor a particular conformation by introducing geometrical constraints. Structure-immunological activity relationship studies concerning how to obtain specific and “superactive” molecules can be achieved by using these novel pseudopeptide analogues. On the other hand, non-natural amino acid residues insertion into peptide chains, have been widely documented and let one infer that modifying the peptide bond can influence the conformational properties and the biological activity of a molecule. These peptide bond isosters and α-carbon asymmetrical analogues belong to a wide family of peptido-mimetic and pseudopeptide molecules.

### Malaria

Malaria, one of the world’s most important public health problems, is a lethal infectious disease resulting in some 300-500 million clinical cases and more than two million deaths per year, mainly among children in developing countries. It is caused by protozoan parasites from the genus *Plasmodium*. The majority of deaths from malaria are due to *Plasmodium falciparum*, the disease’s most virulent causative agent, being its life cycle remarkably complex [1].

Vaccination is considered to be an approach which would complement other strategies for prevention and control of malaria [2,3,4]. A number of parasite molecules from different stages of its life cycle have been tested as vaccine candidates; however, when reproducing artificially the primary structure of the antigen, the resulting native-like molecule has inevitably proven to be poorly immunogenic. Among cell surface proteins, MSA-1 and MSP-2 [5,6,7,8] from blood and CSP from pre-erythrocyte stages, have been extensively studied for their ability to induce protective a immune response against the malaria parasite. Success in vaccine development relies on efforts currently being carried out to identify B as well as T cell epitopes to be further included as possible vaccine candidate antigens.

On the other hand, protein and peptide structures are stabilized by the homogeneous nature of peptide bonds. One L-amino acid is thus bound to another by a Nature-made [CO-NH] amide bond, maintaining the same backbone orientation in a polypeptide chain. However, peptide-based vaccine development is strongly impaired by peptides’ susceptibility to proteolysis, as well as by their predetermined well-codified backbone topochemical spatial arrangements. If a non-polymorphic immunogenic peptide is thus “conveniently, from a pathogen point of view”, constituted by just L-amino acids (all of them bound through naturally-made peptide bonds), it will be subject to *in vivo* peptide bond degradation by proteases when inoculated, as well as being targeted by “smoke screen” induced antibodies as an evasion mechanism used by pathogens. Such non-polymorphic antigens’ chemical code of silence has to be broken by performing strategic chemical modifications, to reveal the selected potential of the immunogens for inducing a specific protection against a given pathogen. These modifications include transforming the α-carbon asymmetrical properties of antigens, as well as the nature of their backbone topology, to allow fine modulated specific fitting into MHC-II molecules, thus provoking a desirable humoral and cell host immune response.

## 2. Peptide Bond Transition State Analogues

**Figure 1:**
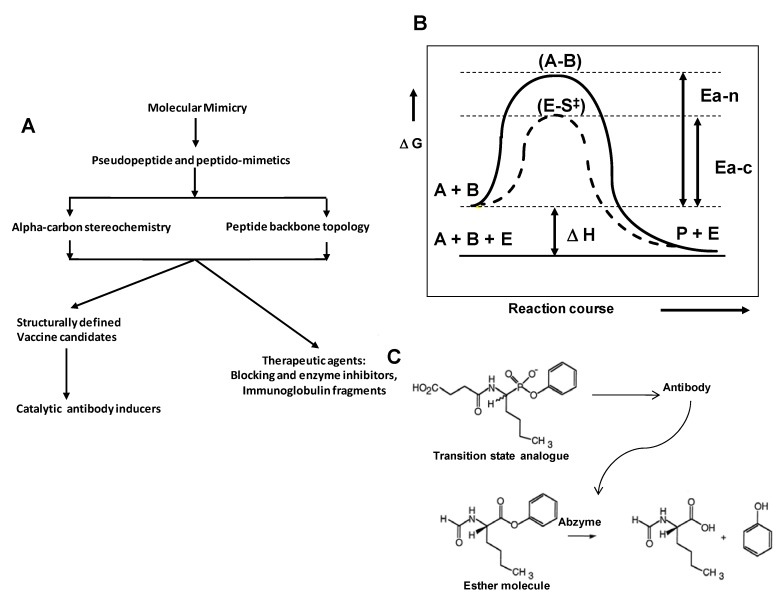
Development of pseudopeptide chemistry. **A.** Applications of pseudopeptide and peptido-mimetics in drug and vaccine discovery. **B.** Energetic profile for a given chemical reaction, being non-catalyzed and processed by a biological catalyst. **C.** Ester hydrolysis reaction catalyzed by antibody 17E8 and structure of the phosphonate transition-state analog used to elicit this antibody as discussed below.

Potential application of pseudopeptides and peptido-mimetics have an important role in generating structure defined molecular probes useful for different applications in immunology, as described in Figure 1A.

### The transition state theory

In enzyme catalyzed reactions, the rate increases while consumed energy decreases, thus when a chemical reaction occurs the energy content of the reacting molecule or atom increases. This is why most chemical reactions whether release or absorb heat, happening faster as the temperature is raised. The high-energy state of the reactants during the substrate transforming process is called “the transition state”**.** Therefore, in a given peptide bond-breaking reaction, the transition state may be one where the reacting bond, although not completely broken, is vibrating at a frequency high enough that it is equally likely that the bonds split apart or reform. Resulting reactants or products produce a loss of energy from the transition state. In consequence, the role of a proteolytic enzyme is to stabilize such transition states while decreasing the energy for the reaction products formation. Enzymes reduce the height of the energetic barrier for achieving the molecular transition state as it was originally proposed by Linus Pauling in the late forties.

In the eighties, Lerner, Benkovich and Schultz postulated that an antibody molecule can be induced by a transition state analogue molecule, in order to be used as a catalyst of a given reaction. Thus, a wide number of monoclonal antibodies were generated and used for catalyzing many chemical reactions, as reference below [9]. Their hypothesis is based on the principle that the transition states’ structures allow direct assessment of the similarities and differences between them and the hapten-molecules designed to mimic them. Subsequent bioinformatics and computational docking studies have been used to garner insight into the processes by which the immune system produces effective catalysts. Therefore, information from such studies can be used to improve catalysis by suggesting alterations to several selected haptens and known antibodies (Figure 1B).

**Scheme 1:**
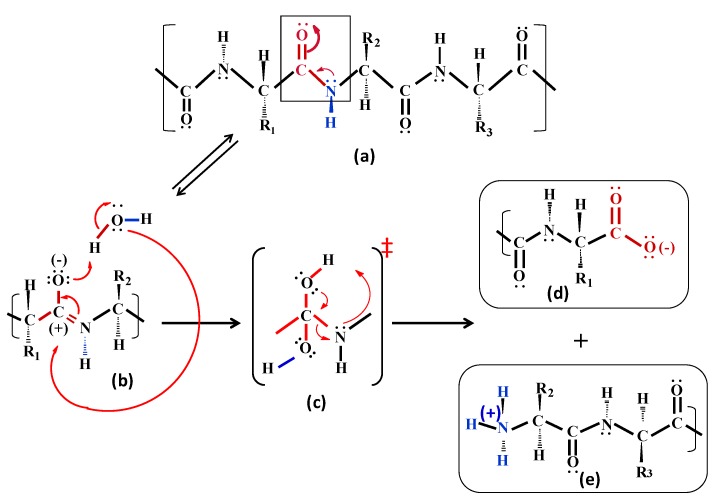
Proposed hypothetical mechanism employed by proteolytic enzymes for substrate hydrolysis.

We have proposed a hypothetical mechanism for peptide bond breakage, as illustrated in Scheme 1. According to the reaction scheme a possible transition state for the peptide bond breaking reaction can be mimicked by used specific site-directed designed pseudopeptides, in which a peptide bond is chemically substituted or modified.

## 3. The Peptide Bond as an Immunotherapeutic Target for Molecule Design

Development of peptide-based vaccines and therapeutic agents has been strongly hampered by their susceptibility to proteolysis, as well as the restrictions implicit in their secondary molecular structure. Preliminary data concerning natural bioactive peptide metabolism normally becomes available shortly after determination of their amino acid sequences. However, information about cleavage sites, hydrolysis chronology and the nature of the protease involved is often incomplete. Most of the time elapsed after degradation is likely to consist of a sequence of relatively non-specific proteolytic events leading to the production of amino acid metabolites. Secondary cleavage sites must be provided or discovered when a first hydrolysis site has not been clearly established or when the chronological cleavage order is not known. On the other hand, a biologically active, soluble linear peptide solution would contain an unpredictable number of peptide conformers; the unique bioactive conformation responsible for such an activity will thus remain quenched by the enormous amount of extra non-structured peptide molecules in solution. Some of them could probably act as antagonists for the bioactive conformational molecule population.

In the first case, two main factors seem to govern a given peptidase’s participation in physiological processes; which are its substrate specificity (dictating the nature of those peptides which can be cleaved) and its cellular location (determining which peptides will be exposed to *in vivo* protease action). Protease identification has progressed over the years, and many observed degradation processes reported in the past are now much better understood due to currently available knowledge that explains formerly unknown endopeptidase activities. However, some of the relevant peptidases are still either unknown or have been insufficiently characterized. In a number of cases, peptide inactivation proceeds from exopeptidase or di-peptidyl peptidase action; for example angiotensins II and III are first degraded by amino-peptidase to become active. It has been well described how proteolytic processing of certain surface proteins from parasites play an important role during infection of a target host cell; blocking such a process could thus be a strategy for avoiding certain parasitic infections [10].

As has been demonstrated, the natural target of protease attack is a particular peptide bond on a peptide substrate; it takes place as a time-function, following molecular recognition and proper folding of both peptide substrate and peptidase [11]. Modified versions of the substrate can be obtained by side-chain amino acid replacement to overcome peptidase specificity for a particular peptide substrate, which would then alter the substrate normal peptidase recognition pattern. A second possibility is direct modification of the target peptide bond, thus inducing backbone alterations on the substrate molecule with subsequent modulation of the peptide three-dimensional structure. Such a peptide surrogate would thus increase its stability against a proteolytic attack, maintaining its biological activity. A pseudopeptide would consequently represent a transition state mimic. Hence, stabilizing specific transition states by mimicking them with structurally defined molecules would have an impact in vaccine development.

### 3.1. Synthesis of reduced amide peptide bond isosters towards exploring the role of backbone malarial antigens and urea-motif containing pseudopeptides

**Scheme 2:**
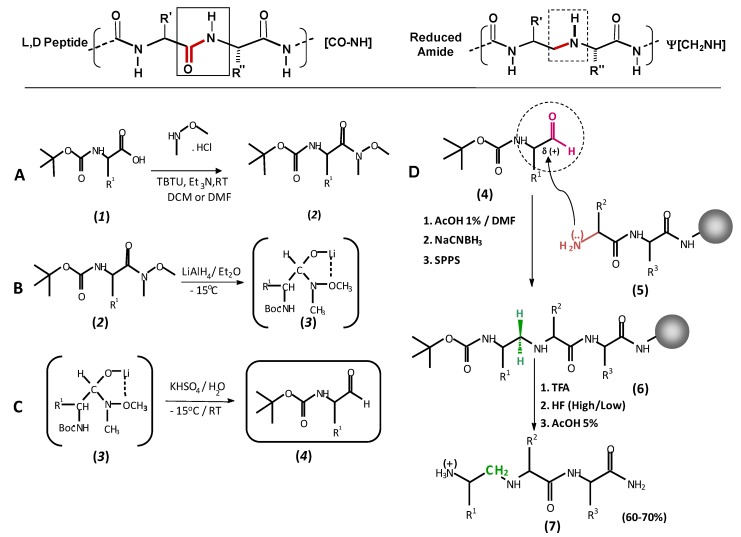
Scheme of solid phase synthesis of reduced amide pseudopeptides. Peptide-bond isoster replacement -[CO-NH]- with ψ[CH_2_–NH]. **A.** Chemical derivatization of an alpha-aminoacid (1) to a carboxamide adduct (2). **B.** Treatment of the carboxamidated product with LiAlH_4_ to produce a non-stable lithium five member ring (3). **C.** An acid treatment produces the target alpha-aminoaldehyde (4). **D.** Solid phase condensation of an alpha-aminoaldehyde (5) to a growing peptide chain (6) and its subsequent alkylative reduction to a methylene amine group (7).

The introduction of peptide bond surrogates (denoted ψ[...], according to Spatola’s nomenclature for pseudopeptides) [12] has been long recognized as a powerful strategy to enhance biological lifetime of peptides, to improve biological activity or selectivity, to design enzyme inhibitors and to induce particular conformational features in potential vaccine candidates. Reduced amide pseudopeptide solid phase synthesis has become an fairly easy procedure to obtain novel structure-defined molecules, as observed in Scheme 2.

**Figure 2:**
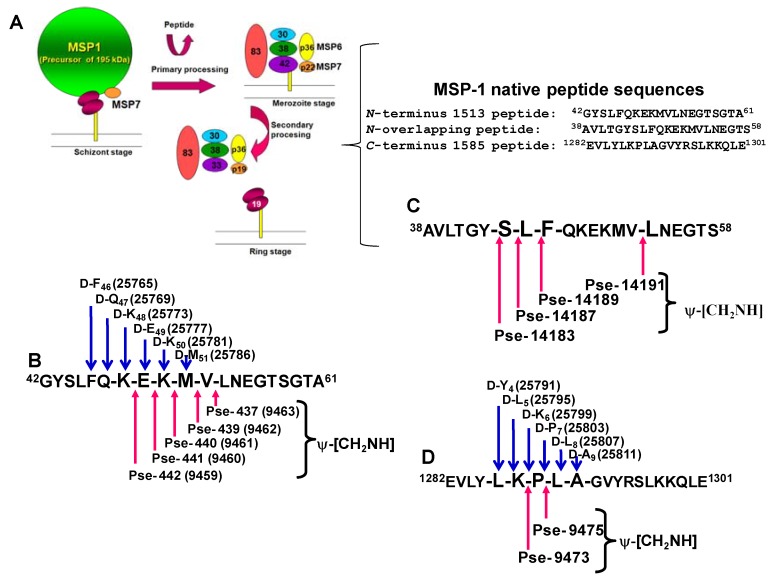
The *Plasmodium falciparum* MSP-1 antigen, as a model for pseudopeptide and peptido-mimetic designing. **A**. MSP-1 proteolytic processing to produces a non-covalent complex on the parasite membrane surface during malarial infection of RBCs. **B.** MSP-1^42–61^ *N*-terminus HABP coded 1513 template sequence, for isoster bond and D-partially substituted analogue design. **C.** MSP-1^38–58^ template sequence and its designed isoster bond analogues. MSP-1^1282–1301^ *C*-terminus sequence HABP coded 1585 template sequence, for isoster bond and D-partially substituted analogue design. Codes for each pseudopeptide were arbitrary assigned to avoid using nomenclature for L- and D- enantiomers. ψ-[CH_2_NH] represents a reduced amide isoster bond.

One of the aims proposed on backbone chemistry is the design and synthesis of oligomeric molecules with unnatural backbones for the formation of novel secondary structures [13] and further applications in biology [14]. In the field of peptido-mimetics, the urea moiety as a non-peptide linkage has attracted much attention in recent years. We have taken different malarial antigens as a model for obtaining a variety of pseudopeptides. First attempts in our molecular design are based on the MSP-1, MSP-2 and other malarial antigens, as can be observed in Figure 2.

It has been described that the MSP-1 antigen suffers two proteolytic events during the process of malaria parasite invasion into RBCs, as observed in Figure 2A. Once identified the specific HABPs used by *Plasmodium* for infecting target cells, and subsequently their critical binding residues, we have performed site-directed replacements peptide bonds associated of given amino-acids for obtaining pseudopeptides. Thus, the *N*-terminus portion of the MSP-1 antigen generated two different families of reduced amide pseudopeptides, as observed in Figure 2B and C, and the MSP-1 C-terminal portion generated another pseudopeptide family, as observed in Figure 2D.

### 3.2. Reduced amide isoster analogues

Reduced amide pseudopeptides are also known as methyleneamino pseudopeptides (Scheme 2). These can be synthesized by different strategies; most of them start by using commercially available *N*-α-protected amino acids. Peptide analogues containing a ψ[CH_2_–NH] reduced amide bond have been investigated during the last two decades within the context of drug and vaccine development, as reviewed here. Several synthetic strategies are discussed elsewhere, but here we will discuss the most useful ways of obtaining such peptide surrogates.

The preferred method for introducing a reduced peptide bond is reductive alkylation of *N-*α*-*protected α-amino aldehydes. A variety of methods have assessed different reducing agents such as H_2_ in the presence of metal catalysts, zinc-acetic acid, borane-pyridine complex, NaCNBH_3_ sodium cyanoborohydride and sodium borohydride acetate NaBH(OAc)_3_, as revised by Guichard [15]; these have been evaluated for reductive alkylation of aldehydes and ketones. A normal procedure for preparing ψ[CH_2_–NH] surrogates involves using NaCNBH_3_ in either acidified methanol or acidified DMF. Nevertheless, NaBH(Oac)_3_ in DCE or THF offers a good alternative for achieving the product. This method for solid phase synthesis strategy was proposed by Sasaky and Coy [16]. As described originally by Sasaky [17], and used in other studies, the reduced amide bond is formed *in situ* on the growing peptide-resin chain; it enhances synthesis speed, since no extensive work up and purification steps are required. This reaction usually proceeds from adding a solution of freshly prepared *N-*protected α-amino aldehyde in DMF containing 1% acetic acid, followed by adding a solution of NaCNBH_3_ in DMF to the peptide-resin growing chain free amino group, where DMF acts as a resin swelling enhancer. The reaction is normally monitored by the ninhydrin test. Secondary amines formed during reductive amination only produces weak coloring on being heated. The chloranil test can be used for the same purpose. If coupling is unsuccessful, it can normally be carried out by using the same procedure, repeated as necessary. Other studies have reported one-pot reductive alkylation of amines by S-ethyl thioesters mediated by triethylsilane and NaBH(Oac)_3_ in the presence of Pd/C. This procedure is particularly useful when the aldehyde is not stable enough to be isolated, but is restricted to solution-phase synthesis (as described earlier) [18].

On the other hand, we have recently proposed an improved method for obtaining *N*-α-*t*-BOC-amino acid aldehydes from asparagine and glutamine for solid phase synthesis of reduced amide pseudopeptides, with the aim of overcoming the difficulties when these amino-acids precursor aldehydes are obtained traditionally in yields lower than 0.5%.

### 3.3. Synthesis of all-retro and all-retro-inverso peptide bond topochemical-surrogates in the exploration of a malarial antigen

**Figure 3:**
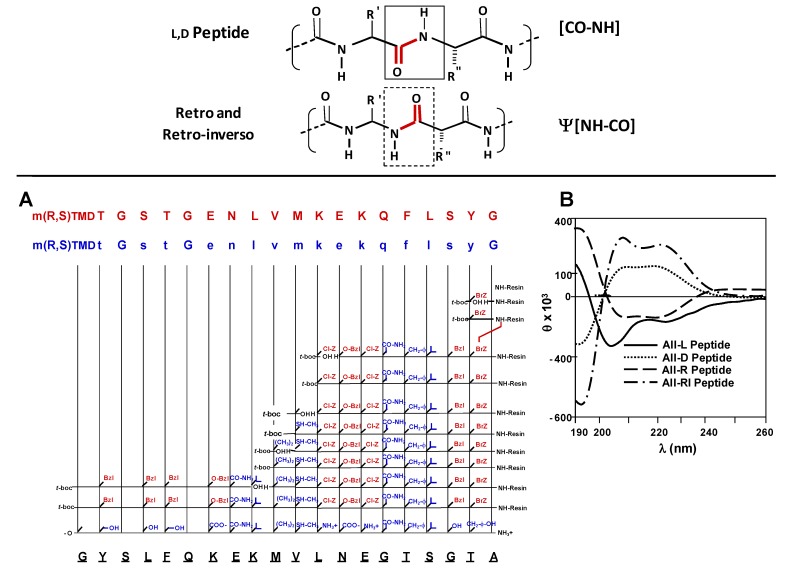
Obtaining all-Retro and all Retro-inverso analogue sequences. A**.** Solid phase synthetical strategy. **B.** Circular dichroism analysis for stereochemical- and topochemical-modified MSP-1^42–61^ pseudopeptides. Overlay of the CD spectra of L, D, Retro, and Retro-inverso MSP-1^42–61^ analogues, small characters represent D-amino acids. Mean residue ellipticity [θ] is shown in degrees cm^2^ dmol^-1^. L-Amino acids are described in capital letters and D-residues as small characters. All-D 1513 (dotted profile), all-Retro analogue (dashed profile); all-D (dotted profile) and all-Retro-inverso (dashed-dotted profile).

As it was nicely reviewed by Michael Chorev, some concepts regarding peptide bond topochemistry, such as the partially modified retro-inverso and end-group modified retro-inverso transformations, were formulated in a joined effort started by Murray Goodman [19]. Consequently a nomenclature describing these novel peptide modifications was established. An experimental design can be observed in Figure 3A. In all-D retro or all-L retro-inverso peptides, the side chains are oriented as in the original L-peptide but the direction of the peptide bonds is reversed (see Figure 3B).

Although the mimicry of L-peptides by all-D retro peptides does not extend to main-chain atoms or to elements of secondary structure, antibodies raised against the L- or the all-D retro peptides cross-react strongly with both structures. Since many B-cell epitopes are located in turns and loops of proteins, there is a considerable potential for achieving antigenic mimicry with all-D retro peptides, as shown with the immuno-dominant MSP-1^38–61^ *Plasmodium falciparum* epitope, which has considerable potential as a synthetic vaccine candidate, as observed in Figure 2C [20].

**Scheme 3:**
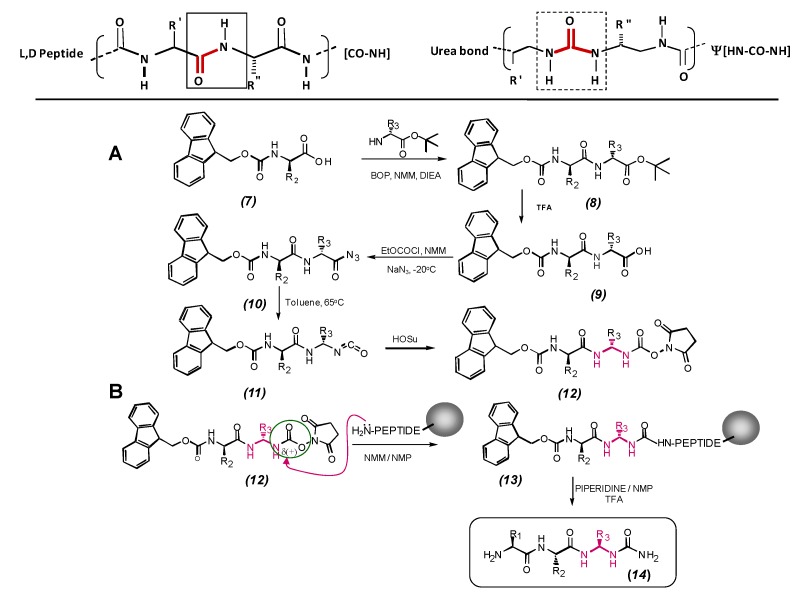
Scheme for solid phase synthesis of urea-bond pseudopeptides. **A.** A Fmoc-*N*-alpha amino-acid (7) is condensed to a *N*-free-*t*-Boc-carbonyl protected amino-acid to give the aduct (8). Thereafter *t*-Boc deprotection, the corresponding carboxylic form (9) is obtained. Curtius rearrangement by EtOCOCl (ethylcloroformiate) in NMM (*N*-methyl morpholine) on (10) gives the product (11). Subsequent reaction of (11) with hydroxysuccinimide (HOSu) produces the target *C*-activated product (12). **B.** Building block (12) is coupled to a *N*-free primary function of a growing chain anchored resin-peptide (13). Successive coupling and deprotection steps using O-succinimidyl carbamates derived from alpha-amino-acids and dipeptides allow the formation of *N,N*´-linked oligoureas (14).

In this context main-chain peptide-mimetics based on peptide-bond reversal and inversion of chirality represent important structural alterations for peptides and proteins, and are highly significant for biotechnology and vaccine development. As will be described later, we have analyzed the antibody cross-reactivity of antibodies induced by a set of MSP-1^42–61^ (1513 peptide-based) reduced amide pseudopeptides against its corresponding D-enantiomer as well as its all-Retro and all-Retro-inverso analogues. Designed molecules are shown in Scheme 3A.

Synthesis procedure to obtain this set of analogues was performed as follow. Regarding the native peptide sequence, the *N*-terminus A61 on all-Retro and all-Retro-inverso analogues was carboxamidated, and a 2-substituted malonic acid (m-*R,S*-TMD) derivative was employed to replace the native *C-*terminus residue as previously reported by Chorev. All-Retro 1513 was synthesized by using L-amino acids and all-Retro-inverso by employing their corresponding enantiomer D-residues. Backbone direction was inverted respecting natural all-L-1513 by anchoring the 2-substituted malonic derivative to the 4-MBHA resin. Thereafter, successive amino-acid couplings were performed as normal, according to standard *t*-Boc procedures reported before for natural L-peptides [21,22]. According to the secondary structure patterns analyzed by CD, in the first set, all-L 1513 (solid black profile in Figure 3B) and its all-D 1513 enantiomer (dotted profile) were seen to be enantiomers because they displayed almost symmetrical CD curves (θ versus λ), but they had opposite signs in the assessed solvent systems, as can be observed in Figure 3B.

Thus, all-L and its all-D 1513 analogue were mirror images of one another. The CD spectrum for the all-L 1513 peptide displayed 208 and 222 nm minimum values and 190 nm maximum ellipticity, characteristic of a right-handed α-helix. The all-D analogue behaved as a left-handed α-helix. The second set compared the all-L 1513 peptide CD profile (solid black profile, lower) with its all-Retro-inverso analogue (dashed black profile, upper); both molecules exhibited a higher CD profile symmetry than in the first case but had opposite signs, behaving as exact mirror images. Both isomer L- and all-Retro-inverso peptides presented a typical helical pattern in TFE solution; such a pattern can be assigned to an α-helix-like conformation having opposite orientation, similar to other studied cases [23]. It is important to bear in mind that the parent all-L 1513 peptide is constituted by L-amino acids, and all-Retro-inverso peptide by D-amino acids; however, the latter has N°C peptide bond orientation, respect to its L-non-modified peptide. The third set contrasted CD curves for the all-D 1513 (dotted profile) with its all-Retro analogue (dashed profile); both displayed quite symmetrical α-helices, but opposite signs, being exact mirror images of each other. The first molecule was made-up of D-amino acids, and the second by L-amino acids but having opposite backbone direction. The fourth set consisted of all-D (dotted profile) and all-Retro-inverso (dashed-dotted profile) peptides, both displaying an α-helix having the same sign but the second being much more α-helical shaped than the first. The fifth set consisted of 1513 all-Retro versus 1513 all-Retro-inverso CD profiles; modifications affected peptide backbone but not side-chain orientation. Their CD curves were also mirror images, reflecting their enantiomeric complementarity. The first was made-up of L-amino acids and the second by D-residues. The all-Retro analogue was thus closely related to its parent all-L 1513 peptide conformation in solution but had opposite peptide bond topochemistry, the same characteristic being displayed when comparing all-Retro-inverso with its all-D analogue.

### 3.4. Succinimidyl carbamate derivatives from N-protected α-amino-acids and dipeptides. Synthesis of ureidopeptides and oligourea peptide pseudopeptides

Several families of peptido-mimetics containing an urea backbone, including *N*,*N-*linked oligoureas [N(CONHR)–(CH2)*m*–]*n*, [4] *N*,*N*-linked oligoureas [NH–CHR–CH2–NH–CO–]*n* [5], ureido-peptoids [NR–CH2–CH2–NH–CO–]*n* [24] and oligomeric imidazolidinones [7], have been described. The urea fragment is promising for drug discovery and biomedical applications because of its expected resistance to protease degradation and interesting H-bonding properties [25,26,27]. In the solid state, *N*,*N*-disubstituted ureas are often involved in self-complementary bidirectional intermolecular H-bonds because of the *trans* geometry of the two amide bonds (*Z*,*Z* conformer) [28,29,30]. Self-organization at the molecular level has also been documented. *N*,*N*-linked oligoureas have be used by Nowick and coworkers to generate intra-molecular H-bonded structures that mimic secondary structural elements of proteins, such as β-sheets and hairpin turns [31]. Moreover, Guichard and coworkers have shown, with a combination of NMR spectroscopy and circular dichroism, that enantiopure *N*,*N*-linked oligo-urea strands bearing proteinogenic side-chains [NH–CHR–CH2–NH–CO–]*n,* as short as seven residues, adopt a stable helical fold stabilized by remote intra-strand bifurcated H-bonds, which is reminiscent of the 14-helix described for the corresponding γ4-peptides [NH–CHR–CH2–CH2–CO–]*n* [32]. The folding predictability of these oligo-urea strands can be integrated with the diversity of available side-chain appendages to develop molecules with specific functions (e.g., antibacterial, amphiphilic, cationic oligoureas) [33]. In this context, modification of peptides by introducing a ureido linkage ψ[NH–CO–NH] between two neighboring α-amino acids is of particular interest. The insertion of a urea fragment between two amino acids has long been seen as a side reaction resulting from an unwanted Curtius rearrangement occurring even at low temperature during peptide coupling with peptidyl and aminoacyl azide derivatives [34]. Three decades ago, Chipens and coworkers first synthesized ureido analogues of biologically active peptides to study their activity [35]. They prepared a number of angiotensin analogues, containing a urea fragment at different positions in the sequence by direct condensation, in solution of a peptidyl isocyanate motif with the free amino group of a second peptide fragment. Interestingly, other applications of ureidopeptides to biology include the synthesis of Leu-enkephalin analogues [36], gastrin antagonists [37] protease inhibitors [38] and a growth hormone with strong *in vivo* activity in the rats [39]. However, little is known about the conformational preferences of pseudopeptides incorporating one or several ureido units [40,41].

Guichard and coworkers started to investigate the intra-molecular H-bonding propensities and conformational behavior of model ureidopeptides in solution [41]. As a result of competitive conjugation, the rotation barriers of substituted ureas are lower than those of its corresponding native amides, and one might expect greater flexibility of the urea linkage in solution. Accordingly, we found that the urea moiety in simple *N*-acyl-*N*-carbamoyl-*gem*-diaminolalkyl residues bearing proteinogenic side-chains adopts a *cis*, *trans* (*E*,*Z*) geometry that can be further stabilized by an intra-molecular H-bond in a non-polar organic solvent. The resulting eight-membered (C8) H-bonded ring has been termed a “urea turn” [41]. With the aim to study in more detail the propensity for urea turn formation in a more comprehensive set of ureidopeptides, including previously unreported oligo-urea/peptide hybrids (made of alternating urea and amide moieties), as well as to incorporate ureido moieties into bioactive peptide segments [42], the group of Guichard have investigated the preparation and reactivity of a variety of succinimidyl carbamates derived from *N*-protected α-amino acids and dipeptides.

Succinimidyl carbamates, which combine both excellent reactivity and stability are useful substitutes for the corresponding isocyanates, which may degrade upon prolonged storage. In addition, we previously reported that linear diamino derivatives (e.g. derived from *N*,*N*-linked oligoureas [NH–CHR–CH_2_–NH–CO–]*n*, or dipeptides) activated at one end as a succinimidyl carbamate, and protected at the other end by a *t*-Boc group, are useful intermediates in the synthesis of cyclic urea-based systems.

### 3.5. Solid-phase synthesis of ureidopeptides and oligo-(amide/urea) hybrids

The Fmoc/*tert*-butyl strategy associated with solid-phase methodology was also considered for the synthesis of ureidopeptides as observed in Scheme 3. Six analogues of the tumor-associated-antigen derived peptide [Leu-27]MART-1(26–35) [43], (H-Glu^26^-Leu-Ala-Gly-Ile-Gly-Ile-Leu-Thr-Val^35^-OH), incorporating one ureido linkage between residues Leu-27 and Ala-28, Gly-30 and Ile-31, Ile-31 and Gly-32, Gly-32 and Ile-33, Ile-33 and Leu-34 or Leu-34 and Thr-35 were selected as targets. They were assembled on a home-made multichannel synthesizer with a semi-automatic mode [44] on a 40 μmol scale starting from Fmoc-Val Wang resin. The formation of the urea linkage was achieved by coupling succinimidyl carbamates, derived from *N*-Fmoc-protected dipeptides (4 equiv.) to the free amino group of the resin-bound peptide in DMF (2 mL) in presence of Hünig’s base (DIEA, 4 equivalents) twice for 120 min [45]. At the end of that time the Kaiser ninhydrin test [46] was negative. The Fmoc group was deprotected with 20% piperidine in DMF, and the last amino acids were introduced with standard solid-phase peptide synthesis procedures. Side-chain deprotection and cleavage of the oligomers from the resin were performed simultaneously by treatment with TFA/H_2_O/triisopropylsilane (95:2.5:2.5) for 60 min at 20 °C. After precipitation in cold ether and centrifugation, crude compounds were purified by RP-HPLC on a C-18 column and analyzed by high resolution mass spectrometry. Again, differential sensitivity of the *N*-acyl-*N*-carbamoyl-*gem*-diaminolalkyl moiety towards acid-catalyzed hydrolysis, depending on the presence or absence of an alkyl side chain, was observed, in agreement with the expectations of Loudon and coworkers for monoacylated *gem*-diamino compounds [47]. Ureidopeptides incorporating a *gem*-Gly moiety are normally recovered in 21% and 13% overall yield after purification, respectively (Scheme 3). Purities of the crude products based on RP-HPLC are also about 75%. However, in cases where molecules containing an alkyl side chain at the *gem*-di-amino moiety, the conditions employed for the molecule and its protecting groups cleavage from the resin, resulted in an extensive hydrolysis of the *N*-acyl-*N*-carbamoyl*gem*-diaminolalkyl moiety, and precluded isolation of the expected ureidopeptides. In some cases, cleavage of resin bound Fmoc-ureidopeptides by TFA treatment afforded truncated ureidopeptide fragments as the major product, resulting from breakdown of the *gem*-diaminoalkyl moiety (65% purity). These results suggest that milder conditions should be employed for safely removing ureidopeptides and oligo (amide/urea) hybrids from the solid supports.

## 4. Immunological Significance of Site-directed Modifications Performed on Malarial Target Antigens

### 4.1. Characterization of a reduced peptide bond analogue from a promiscuous CD4 T cell epitope derived from the merozoite surface protein-1 malaria vaccine candidate of Plasmodium falciparum

The use of synthetic peptides as vaccine components is hampered by their susceptibility to enzymatic degradation and rapid clearance from biological fluids. Introduction of non-natural structural modifications can render the peptides more resistant to enzymatic degradation, encouraging attempts to use such non-natural ligands as components of synthetic sub-unit vaccines. We have compared the antigenic and immunogenic properties of a series of non-natural peptide analogues, derived from a promiscuous T cell epitope of the major *Plasmodium falciparum* malaria vaccine candidate, merozoite surface protein 1 (MSP-1). A series of HLA class II restricted MSP-1^38–58^-specific TCC, established from three volunteers were characterized for their minimal epitope and fine specificity. T cell stimulatory activities of a series of pseudo-peptide analogues with single reduced peptide bond ψ[CH_2_–NH] modifications were compared with those of single D-amino acid replacement analogues. Compared to reduced peptide bond analogues, the single D-amino acid replacement analogues turned out to be less suitable for TCC stimulation. In particular, the reduced peptide analogue carrying a ψ[CH_2_–NH] backbone modification between positions V52 and L53 of MSP-1^38–58^, demonstrated properties that would make it a more suitable vaccine component than the unmodified parent peptide. First, the pseudo-peptide stimulated a number of TCC restricted by a range of HLA class II alleles. Second, trypsin treatment in combination with T-cell stimulation assays, provided evidence of increased resistance to proteolytic digestion. Third, the parasite-binding anti-MSP-1 mAb coded 7.27 recognized better this particular pseudo-peptide in competition ELISA experiments, and its immunogenicity in out-bred *Aotus* monkeys was superior to that of the parent peptide, eliciting antibodies that cross-react with native MSP-1, as described elsewhere [20].

The use of pseudo-peptides for immune response stimulation can offer advantages like immune-modulation [48], resistance to proteolytic degradation [49], mimicking of non-natural B cell epitopes and conversion of non-immunogenic peptides into immunogenic structures [50,51]. MSP-1 is the most studied malaria vaccine candidate from *P. falciparum* asexual blood-stages. MSP-1 is synthesized as a precursor of 185–210 kDa in the late schizont stage, and undergoes post-translational proteolytic processing in several fragments of 83, 42, 38 and 28–30 kDa [52].

Different studies have shown that antibodies against MSP-1 were able to inhibit plasmodial growth *in vivo* and *in vitro,* and immunization of non-human primates with MSP-1 derived peptides lead to partial or complete protection against life challenge [3,53,54]. MSP-1^38–58^ peptide binds to a range of HLA-DR and -DP molecules representing a promiscuous T cell epitope suitable for inclusion into multi-epitope-based vaccines [55,56,20]. The aim of such study was to systematically investigate whether non-natural single D-amino acid replacements and reduced amide ψ[CH_2_–NH] backbone modifications of MSP-1^38–58^ lead to enhanced resistance against proteolysis, while retaining or even improving the antigenic and immunogenic properties of this promiscuous malaria epitope.

**Figure 4:**
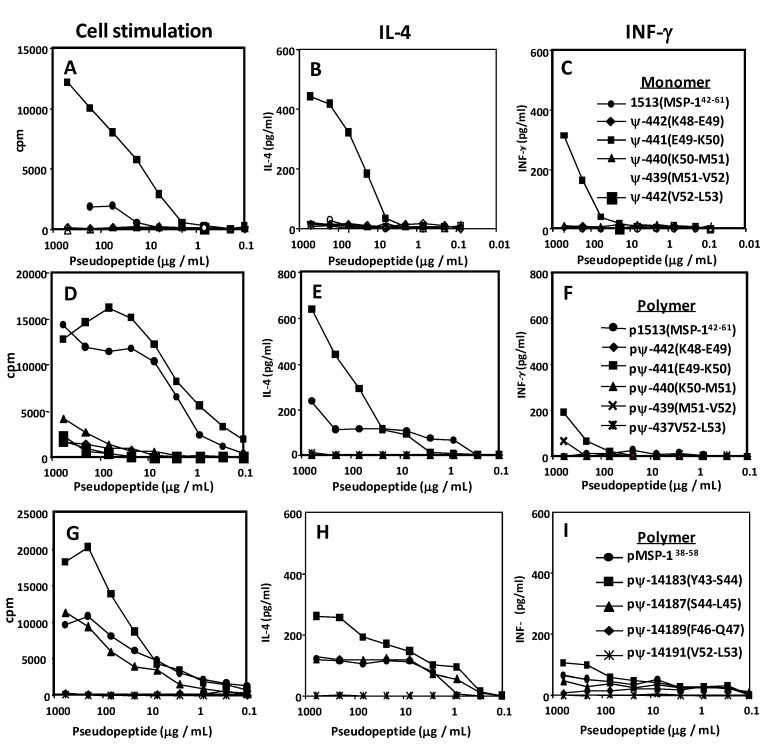
T-cell clone stimulation and cytokine profiles by specific site-directed pseudopeptide analogues of the MSP-1^38–58^ and MSP-1^42–61^ sequences. **A, B** and **C** are stimulation patterns obtained after HLA-DR8 restricted T-cell clone stimulation with MSP-1^42–61^ pseudopeptides. **D, E** and **F** are the stimulation patterns produced after stimulation with MSP-1^38–58^ pseudopeptides.

Nineteen TCC specific for MSP-1^38–58^ and restricted by a range of HLA-DR and -DP molecules were established from three donors immunized with SPf66 in combination with QS21 [55,57]. The minimal epitopes of the TCC were delineated using a series of N- and C-terminally truncated peptides of MSP-1^38–58^. The epitopes varied in length (9–14 residues), and its localization within MSP-1^38–58^ most likely depended on the restriction element and TCR involved in the tri-molecular interaction of APC, peptide and TCR. In 17 of 19 TCC tested, the minimal epitope comprised more than nine amino acid residues. Amino acid positions that are critical for antigen binding and/or T cell activation were mapped using single glycine and alanine amino acid exchange scanning. Amino acid positions not permissive for amino acid residue exchanges are always located within the delineated minimal epitope. Glycine and alanine exchanges are tolerated at the *N*- and *C*-terminal end, outside of the delineated core peptide. This reflects, most likely, the fact that residues flanking the HLA-bound core peptide of nine amino acids are essential for stabilizing HLA-class II-peptide complexes, but are not directly involved in the interaction with the T cell receptor [58,59], as can be observed in Figure 4.

Interestingly, amino acid MSP1-1^38–58^ positions highly sensitive to alanine and glycine exchanges ranged for all TCC tested from Y43 to V52. This stretch of ten amino acids was incorporated into the synthetic peptide vaccine used for immunizing these volunteers, strongly suggesting that the TCC analyzed might be originally primed by vaccination [57].We first compared a series of single D-amino acid replacements and their corresponding reduced amide backbone ψ[CH_2_–NH] modifications in a range of HLA-DR restricted TCC. The results clearly demonstrated that single D-amino acid analogues are less potent in T cell activation compared to pseudo-peptides. Therefore, a new series of derivatives of MSP-1^38–58^ with single reduced amide peptide modifications were tested on a larger TCC panel. Three of the analogues carried single reduced amide peptide bond modifications within the essential epitopes, and one at the *C*-terminal border of the TCC tested. Out of the four derivatives, three could be recognized by one or more TCC, and pseudo-peptide F46*-*Q47 stimulated none of the TCC, confirming results obtained with the alanine and glycine scan, suggesting that F46 and Q47 positions are critical amino acid residues for TCC activation. In contrast, pseudopeptide V52-L53 (ψ-437) was stimulatory for the majority of TCC tested. Comparison of localization of backbone modification tolerated and minimal epitope of TCC showed that analogues are stimulatory when the modification was introduced outside of the critical amino acid positions, mapped with glycine or alanine scanning. If the modification is localized close to critical amino acid positions, no stimulation was observed with pseudo-peptide V52*-*L53 (ψ-437). This interpretation is supported by results obtained with other TCC, that tolerated three out of four backbone modifications and were not sensitive to glycine/alanine exchanges at positions Y43and S44. Some TCR molecules complexed to their antigenic HLA-peptide ligands have been crystallized [60]. The overall topology of the orthogonal docking mode is such that Vα and Vβ domains of the TCR are closest to the *N*- and *C*-terminal residues of the antigenic peptide, respectively [61] and the complementary determining region 1 of the TCR Vβ domain may interact little, if at all, with the bound peptide [60]. These structural features could explain why the pseudo-peptide backbone modifications located at the *C*-terminus of the minimal TCC epitope remained stimulatory for a vast majority of the TCC tested. Secretion of IL-4 or INF-γ by TCC after stimulation with these pseudo-peptides were not modified as has been described in other systems [48], and as observed in Figure 4. The introduction of a single amino methylene replacement resulted in enhanced proteolytic stability of HLA class I restricted peptides [62]. A remote effect of single backbone modifications on the stability of adjacent peptide bonds is known in peptide drug development. Since pseudo-peptides V52-L53 (ψ-437) and S44-L45 (ψ-14187) are presented and recognized in the context of a range of HLA-DR and -DP molecules, we investigated whether the introduction of the amino-methylene bond resulted in increased stability to enzymatic hydrolysis. Trypsin cleaves exclusively peptide bonds in which the carboxyl group is contributed by lysine or arginine residues, regardless of the length or amino acid sequence of the peptide chain. One can estimate the total number of resulting peptide fragments from the number of lysine and arginine residues present. In MSP-1^38–58^, two lysine residues at positions 48 and 50 are present, resulting in two potential digestion sites located in the centre of the minimal epitope of TCC. Pseudo-peptides S44-L45 (ψ-14187) and V52-L53 (ψ-437) were digested with trypsin and resulting stimulation of TCC was assessed by proliferation. Results demonstrated that the introduction of single pseudo-peptide backbone modifications four and two amino acid residues apart from the presumed cutting side of trypsin, significantly enhanced the stability of the analogue. This property is relevant when considering that antigen persistence in biological fluids influence the functional half-life of peptide-HLA complexes, and therefore the immunogenicity of a given structure [62,63,64]. In order to represent a suitable component of a multi-epitope synthetic peptide malaria vaccine, pseudo-peptides should elicit antibodies binding to the *P. falciparum* asexual blood stage parasites. First, it was tested whether pseudo-peptide V52-L53 (ψ-437) is recognized by MSP-1 binding antibodies. mAb 7.27 used in these experiments binds to MSP-1^43–53^, as determined in Western blot analysis, immuno-fluorescence analysis and surface plasmon resonance spectroscopy (Biacore) [65]. Pseudo-peptide V52*-*L53 (ψ-437) competed better, *i.e.* at a lower concentration than the unmodified parent peptide for its binding to mAb 7.27 in competitive ELISA, suggesting that is recognized by this MSP-1 antibody. Next, *Aotus nancymae* animals were immunized with pseudo-peptide V52*-*L53 (ψ-437) to be tested for generation of anti-MSP-1 antibodies. The New World primate *Aotus nancymaae* is susceptible to infection by *Plasmodium falciparum,* and has been recommended by the World Health Organization as a model for malaria vaccine candidate evaluation [66,67]. Out of a group of eight animals, three responded with the production of anti-MSP-1 antibodies, as assessed in Western blot analysis. The major bands of 83 and 72 kDa represent described processing products of the MSP-1 precursor protein [68]. Interestingly, in animals immunized with unmodified parent MSP-1^38–58^ no induction of anti-MSP-1 humoral immune responses were observed, confirming our first observation that native sequences are poorly immunogenic.

In these studies, an antigenic and immunogenic analogue of MSP-1^38–58^ carrying a single amide ψ[CH_2_–NH] bond was identified. The analogue bound to a range of HLA-DR and -DP molecules, showed increased resistance to proteolytic digestion, was recognized by anti-MSP-1 mAb and induced anti-MSP-1 humoral immune responses upon immunization of *Aotus* monkeys. These results support the notion of inclusion of non-natural peptide analogues into the next generation of synthetic peptide-based multi-epitope vaccines [69].

### 4.2. Mapping the anatomy of a Plasmodium falciparum MSP-1 epitope using pseudopeptide-induced mono- and polyclonal antibodies, and CD and NMR conformation analysis

Antigen structure modulation represents an approach towards designing subunit-based malaria vaccines. A specific epitope α-carbon stereochemistry, as well as its backbone topochemistry, were assessed for obtaining novel malarial immunogens. A variety of pseudopeptides derived from the MSP-1^38–61^ epitope of *Plasmodium falciparum* were synthesized, based on solid-phase pseudopeptide chemistry strategies; these included all-L, all-D, partially-D substituted, all-[NH-CO]-Retro, all-[NH-CO]-Retro-inverso, and ψ[CH_2_NH] reduced amide surrogates. We demonstrate that the specific recombinant MSP-1^34–469^ fragment binding to red blood cells (RBCs) is specifically inhibited by non-modified MSP-1^42–61^, as well as by its V52-L53 (ψ-437), M51-V52 (ψ-439) reduced amide surrogates and partial-D substitutions in K48 (25773 analogue) and E49 (25777 analogue). *In vivo* tests revealed that reduced amide pseudopeptide-immunized *Aotus* monkeys induced neutralizing antibodies, specifically recognizing the MSP-1 *N*-terminus region. These findings support the role of molecular conformation in malaria vaccine development.

Conserved *P. falciparum* protein antigens are becoming important targets in the efforts made toward obtaining novel generations of malaria vaccines. In this sense, the role of the *P. falciparum* MSP-1 ligand in the RBC parasite invasion of has been well documented, as well as some of its most relevant binding fragments, such as MSP-1^42–61^, MSP-1^202–221^, and MSP-1^1282–1301^ [70,71,72]. The MSP-1^43-53^ peptide has also proven to be an immuno-dominant epitope when included in malaria vaccine formulations assessed in clinical trials [2,3,4]. MSP-1-derived fragments are thus still regarded as potential subunit-based malarial vaccine components. However, what is not understood are the structural basis for these conserved malaria antigens being neither immunogenic nor protective against this deadly disease.

**Figure 5:**
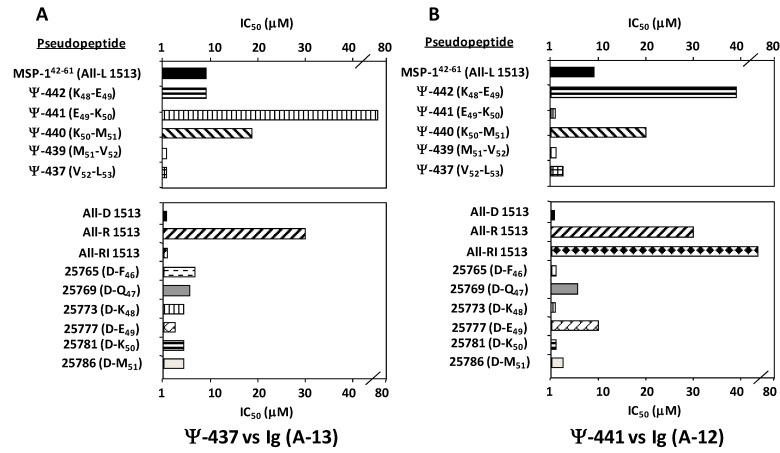
Effects of introducing reduced peptide bonds into MSP-1^42–61^ -derived peptides on purified A-13 and A-12 mAbs molecule binding. Serial dilutions (1-100 nM) of unmodified parent peptide and its pseudopeptide derivatives were incubated with either A-13 in (**A**) or A-12 in (**B**) mAbs in the presence of 4.66 nM ψ-437 and ψ-441 reporter pseudopeptide, respectively. IC_50_ values are given as the average of triplicates from one representative experiment.

On the other hand, we have examined the role of MSP-1^38–61^ stereochemistry, as well as its topological properties, by using a variety of selected pseudopeptides to correlate their *in vivo* and *in vitro* immunological properties. This paper has focused on demonstrating that the all-L 1513 peptide conformer (MSP-1^42–61^), its methylene amino M51-V52 (ψ-439) surrogate, and D-K48 (25773 analogue) and D-E49 (25777 analogue) partial-D-substitutions (these being two residues which are relevant for protein binding) prevented RBC surface proteins to bind specifically to *P. falciparum* recombinant MSP-1^34–469^. The rMSP-1^34–469^ fragment cross-linked to 57 and 18 kDa relative molecular weight band erythrocyte protein groups. *In vivo* tests demonstrated that most *Aotus* monkeys immunized with either of the 1513-derived reduced amide pseudopeptides induced *P. falciparum* merozoite antibody titters. Modifications performed on peptide bonds between K48-E49 (ψ-442), M51-V52 (ψ-439), and V52-L53 (ψ-437) amino-acid pairs, specially induced highly reactive antibody titters to the rMSP-1^34–469^. In contrast, sera reactivity of monkeys immunized with the non-modified 1513 peptide poorly recognized the rMSP-1^34–469^ fragment. Results obtained in this experiment suggest that monoclonal antibodies induced by reduced amide pseudopeptides of the MSP-1^42–61^ epitope, possess chiral selectivity preference by unique-restricted conformations on all-L antigen. The evidence interestingly supports that ψ-439, as well as ψ-437, are adopting specific structural conformations in solution, allowing the peptide backbone to mimic a desired peptide transient structure; this will lead it to interact more efficiently with a chiral paratope stereo-centre on neutralizing mAb A-13 and mAb A-12, as observed in Figure 5. The evidence presented above strongly suggests that a certain folding pattern was present in ψ-439, seeming to correspond to a biologically active conformation recognized by mAb A-12 and mAb A-13. Antibodies raised against the modified reduced amide epitope can specifically recognize those native antigen structural features, which cannot be mimicked by the natural all-L peptide (MSP-1^42–61^ ). Our findings have revealed that receptors on red blood cells for the MSP-1^42–61^ epitope are fairly selective for L-natural conformations on amino-acid ligands, which (as shown), can be mimicked by specific ψ[CH_2_–NH] modifications, inducing a modulated molecular fit. ψ[CH_2_–NH] surrogate antibodies can differentiate topochemistry but not chirality. As a consequence of being highly α-helical, due to strong long distance NOE interaction governing peptide conformation, the non-modified molecule will have two very well-differentiated properties. First, it will fit into its receptors on red blood cells with high affinity, mainly due to the L-restricted chirality of its conforming amino acids; second, it will not fit properly into a proven neutralizing antibody paratope. The rigidity and right-handed properties of the all-L 1513 non-modified molecule will potentially prevent the molecule to induce a protective immune response against malaria. A single reduced amide isoster bond can thus efficiently modulate an entire peptide conformation, mimicking parasite ligand transient-states due to the backbone internal mobility, representing an important aspect to be considered when designing synthetic vaccines. Further application and selection of stereo-specific neutralizing antibodies offers a potential and powerful tool when developing site-directed modifications on targeted pathogen epitopes; this may thus constitute a versatile approach towards designing new malaria vaccines.

### 4.3. MSP-1 malaria pseudopeptide analogs: biological and immunological significance and three-dimensional structure

Merozoite Surface Protein-1 (MSP-1) has been considered as a malaria vaccine candidate. It is processed during the *Plasmodium falciparum* invasion process to RBCs. A conserved MSP-1 *C*-terminal peptide (MSP-1^1282–1301^) was identified as a HABP, known as 1585. Since conserved HABPs are neither antigenic nor immunogenic we decided to assess the significance of a single peptide bond replacement in 1585. Thus, two pseudopeptides were obtained by introducing a ψ[CH_2_–NH] reduced amide isoster into the 1585 critical binding motif. As demonstrated, the pseudopeptides bound to different HLA-DR alleles, suggesting that backbone modifications affect MHC-II binding patterns. Pseudopeptide antibodies inhibit *in vitro* parasite RBC invasion by recognizing MSP-1. Each pseudopeptide-induced antibody shows distinct recognition patterns. ^1^H-NMR studies demonstrated that isoster bonds modulate the pseudopeptides structure, and thus their immunological properties, representing a possible subunit malaria vaccine component.

**Figure 6:**
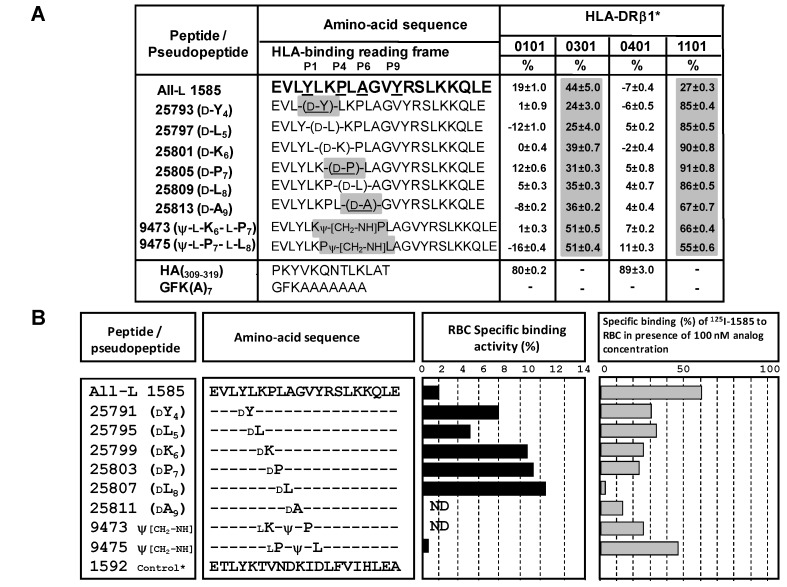
MSP-1^1282–1301^ analogue-HLA-DRβ1∗ alleles and red blood cell-binding patterns. **A.** The relative values for an analyzed peptide binding to a given HLA-allele were determined. Biotinylated peptides used for binding assays were HA: HA306-318; and GFK(A)_7_. Values shown in bold correspond to residues providing efficient binding to a given allele. Values higher than 50% are considered significant and are shaded. **B.** On the center pannel, specific binding activity of ^125^I-all-L 1585 (MSP-1^1282–1301^) peptide and its D-partial substituted analogs to Red Blood Cells (RBCs) in concentrations ranging from 0 to 200 nM; and MSP-1^1282–1301^ (represented by solid bars). Native peptide RBC-binding inhibition by D-partial substituted analogs is shown by gray bands on the right-hand side; ND is used for non-determined and 1592 control* is used for the 1592 (MSP-1^1422–1441^) non-binding peptide.

Our findings clearly support the unique properties of this pseudopeptide family. Peptide bonds used in Nature to built proteins can be replaced, mimicking possible transient molecular conformations which peptides cannot achieve on their own. This study demonstrates that a single peptide bond modulates the peptide entire 3D structure, and thus its immunological properties when it is replaced by a ψ[CH_2_–NH] isoster bond. Peptide 1585 is poorly immunogenic, probably due to its structurally well-organized helical conformation, as previously reported [71], which would allow this peptide backbone to adopt a more suitable HLA-interacting conformation, as observed in Figure 6A.

This will allow strong molecular-docking in the HLA-DRB1* 0102 molecule, but subsequently avoid an appropriate orientation to TCR contact residues. 1585 pseudopeptide analogs (having restricted conformational features) will allow not only appropriate docking in the HLA-DR molecule, but better orientation to TCR contact residues. ψ[CH_2_–NH] isoster containing pseudopeptides improved interaction with the 15 conserved H bonds established by HLA-DR molecules α and β chains. These have an appropriate peptide backbone that induces TCR contact residue orientation [73-76], acting like an altered peptide ligand [74]. The molecules used in this study only differed in one peptide bond, as the amino acid sequence was identical for all of them.

Pseudopeptides should thus have a binding tropism for one HLA-DR molecule, depending on the specific peptide bond replacement, and in turn depending on that pseudopeptide reading-frame established from a particular peptide. Since these pseudopeptides induced sequence location- dependent changes in peptide conformation, binding to MHC molecules with different patterns compared to wild-type peptide, were perceived by TCR in a subtly different way as stimulating some TCR effector functions. Our results provided evidence that peptide bond modifications transform an antagonist peptide into an agonist, inducing an immune response linked to an individual’s genetic background. The peptide backbone greatly influences molecule reading-frame interaction with the MHCII-TCR complex, thus selecting the type of immune response to be elicited (Figure 6A).

On the other hand, we have demonstrated that 1585 peptide D-analogue are specifically bound to RBCs. Thus, ^125^I-labeled 1585 peptide and its D-surrogates specifically bound to RBC membrane proteins in absence of their corresponding non-radio-labeled 1585 analog peptides. Specific binding was inhibited by the presence of the non-radio-labeled molecules. In these experiments, total binding refers to the quantity of labeled peptides bound to the erythrocytes in absence of unlabeled ligand; non-specific binding is the amount of labeled peptides bound to cells in the presence of an excess of identical non-labeled peptide. Specific binding is the difference between total and non-specific binding.

If the relationship between the quantity of ^125^I-peptide which binds specifically and the quantity of ^125^I-peptide added initially gave values >2% (or 0.02 pmol bound, per pmol added), at the point where there is a straight correlation between the concentration and binding activity, the peptide was then considered to be a high binding one.

As can be observed in Figure 6B, all-L 1585 parent peptide displayed high binding activity to RBCs; however, proposed D-substitutions have proven to be more active than the parent molecule (as shown in black bars), with D-substitutions in K6, P7, and L8 being responsible for higher binding activity. Accordingly, on the right-hand side of Figure 6B, gray bars shown that specific ^125^I-all-L 1585 binding is totally avoided at 100 nM D-surrogate concentration by the 2587 analog (D-L8). D-Substitutions in residues K6, P7, and A9 were also shown to be relevant for binding of this MSP-1 fragment to RBCs.

**Figure 7:**
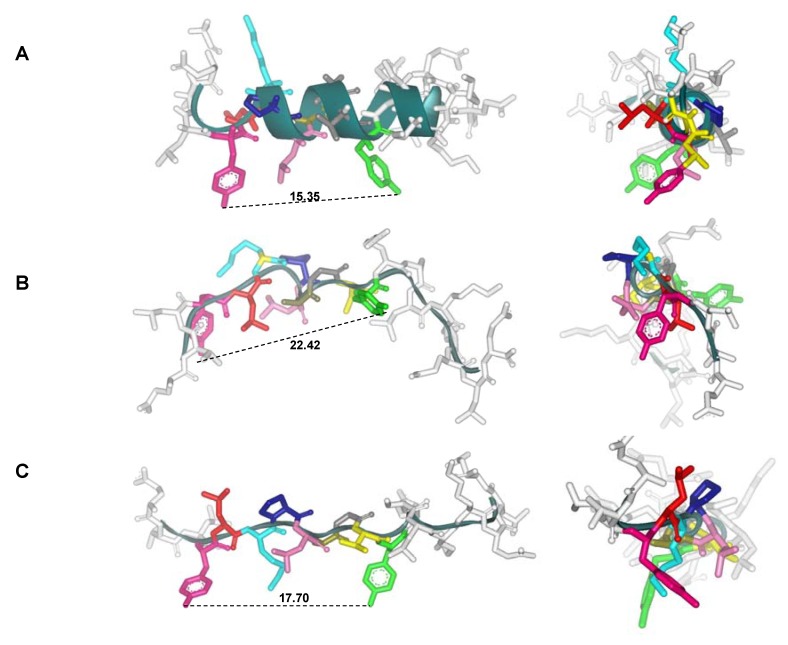

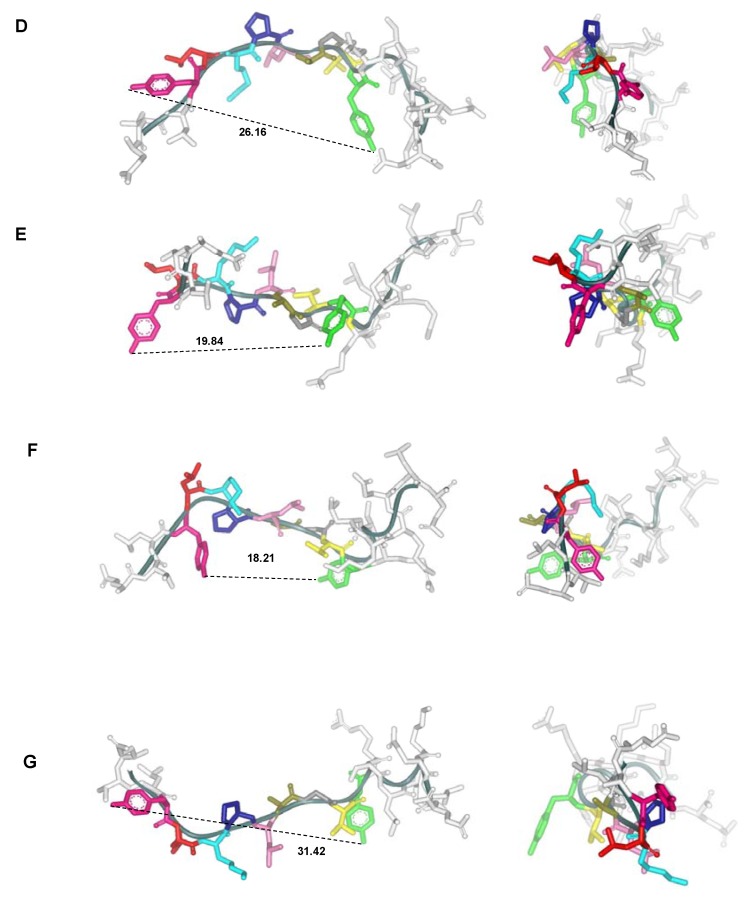
Conformational effects induced by D-substitutions in the all-L 1585 peptide 3D structure. The most representative structures for the parent all-L 1585 peptide and its D-partial substituted analogs and a reduced amide analog are analyzed respecting their 3D structure. Molecular distance between Y4 and Y12 is shown in Å. **A.** Native MSP-1^1282–1301^ (1585 peptide). **B.** 25793 (DY4). **C.** 25797 (DL5). **D.** 25801 (DK6). **E.** 25805 (DP7). **F.** 9473 (LL6-ψ[CH_2_NH]-LP7). **G.** 9475 (LP7-ψ[CH_2_NH]-LL8).

On the other hand, BALB/c mice immunized with the double-modified analogs (D-amino acid-ψ[CH_2_–NH]-L amino acid), specifically in L5, K6, and A9 residues, strongly recognized a rMSP-1^1250–1563^ fragment (Figure 7). Such double-modified analogs displayed immunological properties for discovering structural features of the protein fragment, as judged by the observed antigen–antibody reactivity. Antibodies induced by these peptides recognized specific structures in the MSP-1 protein. Accordingly, sera from the same immunized mice specifically recognized the 42 and 33 kDa cleavage products containing the MSP-1^1282–1301^ amino acid sequence. These data confirm that the parent all-L 1585 peptide is poorly immunogenic, as reported before [71], while some of its peptide modifications render this MSP-1 sequence immunogenic. Therefore this, shows for the first time how a simultaneous single asymmetrical transformation in only one α-carbon and a site-directed backbone modification contributed to strong recognition of structural features present in the 42 and 33 kDa MSP-1 cleavage fragments (Figure 7). This also contributed to strong reactivity against a MSP-1^1250–1563^ recombinantly expressed fragment by mouse antibodies induced by this analog (D-A9). Hence, the adjacent peptide bonds to A9 and its a-carbon asymmetry are crucial for proper humoral response stimulation against the *P. falciparum* malaria parasite. Additional immunological activity assays in *Aotus* monkeys, for this group of doubly modified pseudopeptides, are currently in progress. This fact could be partly due first to an increase in the distance between Y4 and Y12 (putative reading frame) in this D-analog which was 26.16 Å, and second to being part of a modified a-helical molecular 3D structure, as shown in its CD pattern and NMR-obtained 3D structure. No other D-substitutions in this amino acid sequence induced an immuno-protective effect against malaria, except for the isoster bond modification between LP7-ψ[CH_2_–NH]-LL8 which was responsible for protection in one out of ten immunized monkeys.

This pseudopeptide had a Y4–Y12 distance of 26.09 Å. In spite of the fact that three out of eight immunized monkeys produced antibody titers higher than 1:80, one of those monkeys partially controlled malarial infection, displaying low levels of parasitemia even after the 14th day following parasite challenge (Figure 7).

All monkeys immunized with the peptide D-L8 analog became infected with malaria at the same rate as the first group immunized with the non-modified parent molecule; only two out of 10 monkeys produced antibodies, although at low titers. This is associated with a shorter distance between the same residues (19.84 Å), being quite close to the native all-L 1585 peptide (15.35 Å), as observed in Figure 7. The present data allowed us to suggest that a distance of around 26.16 Å may be in the range of distances between those peptide amino acids required to fit into pockets 1 and 9 of any HLA-DRβ1* molecule in immunogenic and protection-inducing modified conserved HABPs. Thus, very compact and rigid peptides having lesser distances, such as the one observed for the all-L 1585 peptide, or those having not-appropriately orientated side-chains (*i.e.,* 25807, D-L8 analog) with respect to the protection-inducing one, will not fit well into Class-II molecules, and thus not allow MHC-II–peptide–TCR complex formation for activating properly the immune system. This work has demonstrated that a single site-directed a-carbon asymmetry transformation in the all-L 1585 peptide ^1286^LKP-A^1290^-binding motif contributed towards specific binding to human HLA-DRβ1*1101 allele, represented in the *Aotus* monkey as *Aona*-DRβ*11, which is known to have very high homology with the human HLA-DRβ* 08, 11, 12, 13, and 14 allele complex [77]. Hence, an asymmetrical rotation of 109.5 Å respect to the plane of a specific amino acid side-chain, has proven to be crucial for a proper MHC-peptide-TCR complex, thus triggering immune partial protection against malaria. Consequently, strategically replacing one or more selected amino acids at a time in a given non-immunogenic peptide, renders it more immunogenic and a protection inducer against malaria, which can be directly associated with binding to specific HLA-DRβ* alleles, as demonstrated in this experiment.

Evidence thus provided in this work, reveals for the first time that the α-carbon asymmetry in D-containing peptides modulates molecular conformation by inducing some flexibility, and so modulating helical regions in the target molecule to be modified. This therefore produces new biological properties in the resulting analogs, such as better fitting into HLA-DRβ1* molecules as shown, and hence induces a protective immune response against malaria, providing new clues in developing a site-directed, modified sub-unit-based, multi-component, synthetic malaria vaccine

### 4.4. Antibodies induced by Plasmodium falciparum merozoite surface antigen-2-designed pseudopeptides, possess neutralizing properties of the in vitro malarial infection

**Figure 8:**
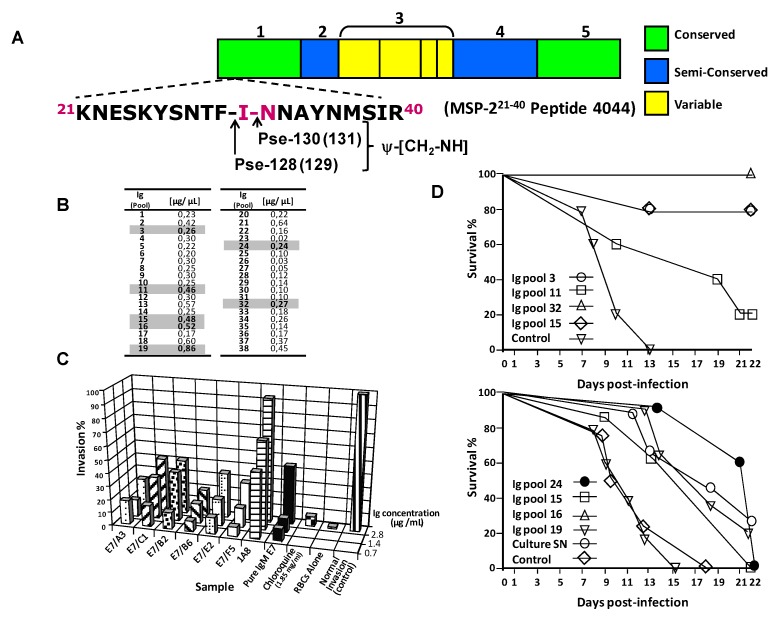
MSP-2 immunoglobulins passively transferred to BALB/c mice. **A.**Genetic organization of the MSP-2. **B**. Ig used for all experiments. **C**. *in vitro* malarial invasion inhibition experiments. **D**. Survival percentage by passive transfer of Ig and challenged with a dose of 5 × 10^3^ iRBC (upper panel). Survival percentage by passive transfer of Ig and challenged with a dose of 2.5 × 10^4^ iRBC with a *Plasmodium berghei ANKA* strain (lower panel).

Pseudopeptide chemistry is gaining ground in the field of synthetic vaccine development. We have previously demonstrated the potential scope of introducing reduced amide peptide bond isosters in a site-directed design, to obtain structurally modified probes able to induce malaria infection-neutralizing antibodies derived from the MSP-1 antigen. We have also reported in another work the functional properties of polyclonal and monoclonal antibodies induced by site-directed designed MSP-2 *N*-terminus pseudopeptides, and their capacity for antibody isotype switching in *in vitro* immunization. Structural properties of the native peptide and its pseudopeptide analogs are discussed, within the context of these novel pseudopeptides induced monoclonal antibody functional and physical-chemical properties, as observed in Figure 8A. On the other hand, as observed in the results, both pseudopeptide molecules induced higher antibody titers in all immunized monkeys, while animals immunized with the native non-modified sequence induced a poor humoral response. This finding is in total agreement with the poor immunogenicity of the native sequence previously reported [78].

As a consequence of the immunological potential of these reduced amide pseudopeptides, a monoclonal antibody directed against the MSP-2^21–40^ was produced (Figure 8B and C). This monoclonal isotype was originally consisting with an IgM, it was then switched to IgG1 by immunizing *in vitro* the hybridoma with the ψ-130 surrogate. Remarkably, both the IgM and IgG1 strongly recognized two bands, having electrophoretical relative mobilities of 97 and 30 kDa, respectively in *P. falciparum* merozoites.

A band about 45 kDa is also detected in mature schizonts. The latter is in agreement with the data reported elsewhere, while the former can be assigned to a dimer form of the protein. Outstandingly, the produced antibody displayed neutralizing properties to the RBC malaria infection, by inhibiting *in vitro* the malaria invasion in 90% in presence of its IgM isotype, and about 40% for IgG1. In order to understand the significance of the immunological and biological behavior of the induced MSP-2^21–40^ pseudopeptide antibodies, the *in vitro* affinity demonstrated that there is a specific interaction between the amino acid sequence when a peptide bond isoster is present, probably allowing a better antigen–antibody fitting for the molecular recognition. On the other hand, mice passively transferred with Ig against both ψ-128 and ψ-130 became selectively protected after being challenged with two different iRBC doses with *Plasmodium berghei*.

### 4.5. Passive transfer of Plasmodium falciparum MSP-2 pseudopeptide-induced antibodies efficiently controlled parasitemia in Plasmodium berghei-infected mice

We have developed monoclonal antibodies directed against the pseudopeptide ψ-130, derived from the highly conserved malarial antigen *Plasmodium falciparum* merozoite MSP-2, for the purpose of obtaining novel molecular tools with potential applications in the control of malaria. Following isotype switching, these antibodies were tested for their ability to suppress blood-stage parasitemia through passive immunization in malaria-infected mice. Some proved total effectiveness in suppressing a lethal blood-stage challenge infection, and others reduced malarial parasitemia. Protection against *Plasmodium berghei* malaria, following Ig passive immunization can be associated with specific immunoglobulins induced by a site-directed designed MSP-2 reduced amide pseudopeptide, as can be seen in Figure 8D. This study proved that antibodies produced by mAb E7, B9 and C1 were capable of inhibiting parasite *in vitro* and *in vivo* invasion, regardless of the immunoglobulin isotype proportion present in each studied Ig pool. It would thus be interesting to find out whether there are any differences between protection achieved by passively immunizing with a specific isotype, and whether interaction between different isotypes produced a synergistic effect, as has been recently reported [79,80]. The results presented herein provide for the first time clear evidence of the existence of a *P. falciparum* MSP-2^21–40^ homolog sequence in the *P. berghei* genome, since mice being challenged with the heterologous *P. berghei*-ANKA strain were capable of efficiently controlling and eliminating malarial induced infections.

Additionally, structurally modified antigens, constituted by *Plasmodium falciparum* reduced amide pseudopeptides are efficient antibody inducers capable of neutralizing malarial infection by blocking specific pathogen ligands. The therapeutical and neutralizing power of any of the MSP-2-pseudopeptide-antibodies could be attributed to individual Ig structural properties. Altogether, the results presented in this work provide some evidence confirming that selected site-directed designed pseudopeptides becomes a reliable strategy towards obtaining novel and versatile molecular tools for a sub-unit, multi-component anti-malarial vaccine formulation, having a potential immuno-therapeutic application. Our molecular designing propose pseudopeptides as potential inducers of catalytic antibodies specific for Plasmodium relevant antigens in agreement with some ideas elsewhere discussed [81].

### 4.6. Antimalarial protection is conferred by passively transferring rabbit F(ab)2’ antibody fragments induced against Plasmodium falciparum MSP-1 site-directed modified pseudopeptides in a rodent model

F(ab)_2_’-immunoglobulin (Ig) fragments induced by site-directed designed immunogens are emerging as novel tools potentially useful in the treatment of transmissible diseases clinical episodes, such as malaria. Immunogens based on reduced amide pseudopeptides based on site-directed molecular modifications represent structural probes that could be considered as novel vaccine candidates, as we have previously demonstrated.

**Figure 9:**
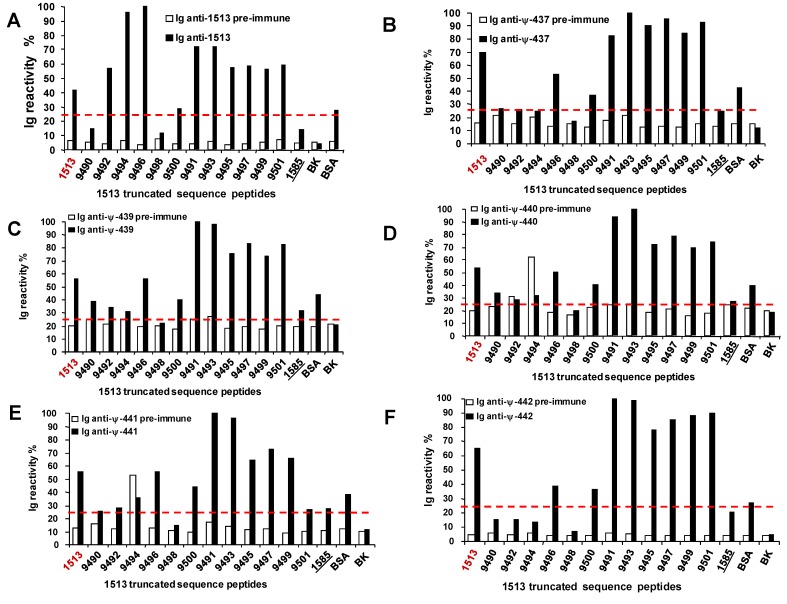
Mapping of 1513-pseudopeptide induced antibodies by competition ELISA. Ig reactivity % in all cases was normalized by selecting the highest optical density at 450 nm as the 100% reactivity. The threshold was arbitrarily established as 25% (dotted red lines).**A.** Reactivity of polyclonal antibodies induced by the entire native peptide 1513 sequence (in red) (MSP-1^42–61^ ). **B.** Reactivity of polyclonal antibodies induced by the pseudopeptide ψ-437 (Val-Leu). **C.** Reactivity of polyclonal antibodies induced by the pseudopeptide ψ-439 (Met-Val). **D.** Reactivity of polyclonal antibodies induced by the pseudopeptide ψ-440 (Lys-Met). **E.** Reactivity of polyclonal antibodies induced by the pseudopeptide ψ-441 (Glu-Lys). **F.** Reactivity of polyclonal antibodies induced by the pseudopeptide ψ-442 (Lys-Glu). In all cases peptide 1585 was employed as a non-relevant sequence, BSA as control and PBS as blank.

**Figure 10:**
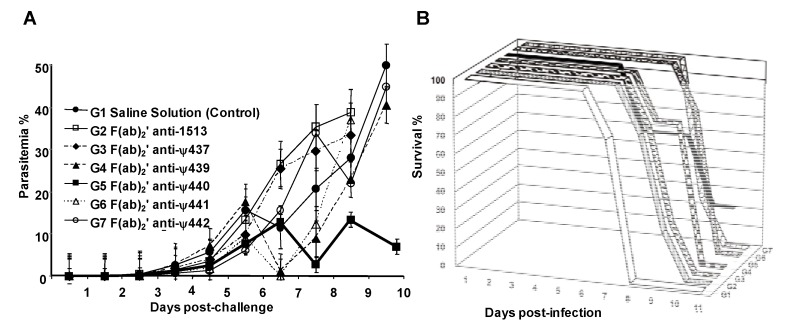
Course of parasitemia in BALB/c mice inoculated with different doses of *P. yoelii-*infected RBCs. **A.** Infection dose: 5 × 10^4^ iRBCs. **B.** Infection dose: 25,000 iRBCs. Each line represents the mean parasitemia of each animal group with its corresponding SD. **C**. Parasitized mouse RBCs. **D**. Mouse 18 protected against malaria by passive transfer of F(ab)_2_’ fragments induced against pseudopeptide ψ-440 (Lys^50^-Met^51^) was able to completely clear out malaria parasites.

We have obtained F(ab)_2_’-Ig rabbit antibodies induced against the *N*-terminal sequence of the native MSP-1 of *Plasmodium falciparum,* and a set of five MSP-1-derived reduced amide pseudopeptides. Pseudopeptides were designed for inducing functional neutralizing mono-specific polyclonal antibodies, with potential applications in the control of malaria. Remarkably, antibodies directed to modified-isoster analogues are mainly directed to the *C*-portion of the native primary sequence (parent 1513), as observed in Figure 9. According to our results, the consensus sequence that better represents this possible B-epitope recognized by those induced antibodies is ^51^MVLNEGTSGTA^61^ or MSP-1^51-61^. In all performed antigenicity tests, Igs present in sera samples poorly recognized the peptide coded 1585 (MSP-1^1282–1301^) as a non-relevant sequence. Following a classical enzyme immunoglobulin fractionation, F(ab)_2_’-Ig fragments, were tested for their ability to suppress blood-stage parasitemia by passive immunization in malaria-infected mice. Some of these fragments proved totally effective in suppressing a lethal blood-stage challenge infection and others reduced malarial parasitemia. Parasitemia profiles and infected-animal survival percentages can be observed in Figure 10A and B.

These data suggest that protection against *Plasmodium yoelii* malaria following passive transfer of structurally well-defined β-strand F(ab)_2_’-Ig fragments, can be associated with specific immunoglobulins induced by site-directed designed MSP-1 reduced amide pseudopeptides.

## 5. Conclusions

Malaria is responsible of high mortality levels, and each 30 seconds a child from an endemic area dies because of malaria. Constituting one of the most important problems in public health, it is still gaining importance among all transmissible diseases, mainly due to the fact that it has a negative impact on the economy and public health; in addition it is expanding through the World. Consequently, the search for potent synthetic vaccines and novel therapeutical strategies for controlling malaria remains an urgent need. Among these novel strategies, the design of potential bioactive molecules in a site-directed fashion, will allow us to obtain highly specific antibody reactivity directed to efficiently block specific pathogen ligands. This new kind of molecular weapon should be able to induce neutralizing antibodies, having strong functional properties to avoid the *in vivo* infection and the subsequent disease. In a previous works, we have obtained evidence that indicates that reduced amide pseudopeptides are structure-defined probes capable of modulating the host humoral immune response, by inducing Ig having neutralizing properties on malaria infection. Remarkably, those animals that has been passively transferred with either an entire Ig molecule or its derived F(ab)´_2_ fragments and infected with rodent malaria, can control the infection by delaying the appearance of parasitemia, and remarkably, most of them become fully protected, evidencing a specific antigen-antibody specific interaction. In our research we have demonstrated that a single methylene amine ψ[CH_2_NH] isoster bond replacement in the target malaria antigen have specifically directed the antibody stimulation reactivity to the *C*-terminal portion of the parent antigen amino-acid sequence, revealing possible unknown B-epitopes, for example the MSP-1^51-61^ sequence and its geometrically defined induced antibody F(ab)_2_’ fragments conferred a precise parasite blocking effect.

The therapeutical and neutralizing power of site-directed designed pseudopeptide-antibodies could be attributed to individual Ig structural properties. In consequence, passively transferring any specific F(ab)_2_’-antibody fragment, constitutes also a potential therapeutical strategy to be further considered in the treatment of malaria infections.
